# The JAK1/2 inhibitor ruxolitinib in patients with COVID-19 triggered hyperinflammation: the RuxCoFlam trial

**DOI:** 10.1038/s41375-023-01979-w

**Published:** 2023-07-28

**Authors:** J. Hammersen, S. Birndt, K. Döhner, P. Reuken, A. Stallmach, P. Sauerbrey, F. La Rosée, M. Pfirrmann, C. Fabisch, M. Weiss, K. Träger, H. Bremer, S. Russo, G. Illerhaus, D. Drömann, S. Schneider, P. La Rosée, A. Hochhaus

**Affiliations:** 1 Universitätsklinikum Jena, Klinik für Innere Medizin II, Hämatologie und Internistische Onkologie, Jena, Germany; 2 Universitätsklinikum Ulm, Klinik für Innere Medizin III, Hämatologie, Onkologie, Palliativmedizin, Rheumatologie und Infektionskrankheiten, Ulm, Germany; 3 Universitätsklinikum Jena, Klinik für Innere Medizin IV, Gastroenterologie, Hepatologie, Infektiologie, Interdisziplinäre Endoskopie, Jena, Germany; 4 Medizinische Fakultät Carl Gustav Carus, Technische Universität Dresden, Dresden, Germany; 5 Institut für Medizinische Informationsverarbeitung, Biometrie und Epidemiologie (IBE), Medizinische Fakultät, Ludwig-Maximilians-Universität München, München, Germany; 6 Universitätsklinikum Ulm, Klinik für Anästhesiologie und Intensivmedizin, Ulm, Germany; 7 Schwarzwald-Baar Klinikum, Lungenzentrum Donaueschingen, Donaueschingen, Germany; 8 Schwarzwald-Baar Klinikum, Klinik für Anästhesiologie, Intensiv-, Notfall- und Schmerzmedizin, Villingen-Schwenningen, Germany; 9 Klinikum Stuttgart, Klinik für Hämatologie, Onkologie, Stammzelltransplantation und Palliativmedizin, Stuttgart, Germany; 10 Universitätsklinikum Schleswig-Holstein, Medizinische Klinik III, Pulmologie, Lübeck, Germany; 11 SRH Klinikum Gera, Klinik für Pneumologie/Infektiologie, Hämatologie/Onkologie, Rheumatologie, Gera, Germany; 12 Schwarzwald-Baar Klinikum, Klinik für Innere Medizin II, Hämatologie, Onkologie, Immunologie, Infektiologie und Palliativmedizin, Villingen-Schwenningen, Germany

**Keywords:** Diseases, Immunology

## Abstract

Dysregulated hyperinflammatory response is key in the pathogenesis in patients with severe COVID-19 leading to acute respiratory distress syndrome and multiorgan failure. Whilst immunosuppression has been proven to be effective, potential biological targets and optimal timing of treatment are still conflicting. We sought to evaluate efficacy and safety of the Janus Kinase 1/2 inhibitor ruxolitinib, employing the previously developed COVID-19 Inflammation Score (CIS) in a prospective multicenter open label phase II trial (NCT04338958). Primary objective was reversal of hyperinflammation (CIS reduction of ≥25% at day 7 in ≥20% of patients). In 184 patients with a CIS of ≥10 (median 12) ruxolitinib was commenced at an initial dose of 10 mg twice daily and applied over a median of 14 days (range, 2–31). On day 7, median CIS declined to 6 (range, 1–13); 71% of patients (CI 64–77%) achieved a ≥25% CIS reduction accompanied by a reduction of markers of inflammation. Median cumulative dose was 272.5 mg/d. Treatment was well tolerated without any grade 3–5 adverse events related to ruxolitinib. Forty-four patients (23.9%) died, all without reported association to study drug. In conclusion, ruxolitinib proved to be safe and effective in a cohort of COVID-19 patients with defined hyperinflammation.

## Introduction

A significant minority of patients with SARS-CoV-2-infection may suffer from severe lung injury, acute respiratory distress syndrome, and multiorgan failure [1, 2]. Treatment and prevention of this fatal clinical course led to a global crisis of health systems and economy [3, 4]. Risk factors, such as virus variants, obesity, comorbidity, age, immunosuppression, gender, and vaccination status were specified but are not predictive markers for clinical decision making [1, 5]. Early signs of inflammation exceeding the expectable levels of a respiratory virus infection have been proposed as predictors of severe courses [6–8]. Prior to clinical deterioration, sudden rise of cytokine levels has been reported, referred to as “cytokine storm”. This results in the so-called hyperinflammation, an overdriven unselective immune response that may severely damage the host [6, 9–11]. Hyperinflammation describes a condition of imminent or actual organ failure caused by dysregulated release of inflammation mediators in response to SARS-CoV-2-infection leading to the severe clinical course of COVID-19 [11, 12]. Affected patients frequently require anti-inflammatory treatment beyond glucocorticoids [13–15]. In this context, inhibition of the proinflammatory JAK/STAT pathway was considered a promising approach to be investigated [16]. Consecutively, the Janus kinase (JAK) 1/2 inhibitor baricitinib was approved for treatment of COVID-19 in 2021 [17, 18]. Simultaneously, efficacy of ruxolitinib was tested in several stages of COVID-19 [19–25]. Ruxolitinib is a potent and selective inhibitor of Janus kinases (JAK) 1 and 2, with modest to marked selectivity against tyrosine kinase (TYK) 2 and JAK3, respectively.

After positive evaluation of the treatment of individual patients [26] we sought to investigate the clinical benefit of ruxolitinib prospectively in patients with severe hyperinflammation as defined by the recently proposed Covid-19 Inflammation Score (CIS) in a large cohort of patients. Based on the experience in hemophagocytic lymphohistiocytosis (HLH), the score was designed to define a cohort of patients who require specific inhibition, and to reflect changes of dynamic parameters (such as fever, ferritin, organ damage, reversal of coagulation disturbance, cytokine suppression) [26].

## Patients and methods

### Study design

RuxCoFlam (NCT04338958) was a single arm, non-randomized open phase II trial for frontline treatment of adult patients with SARS-CoV-2 induced defined hyperinflammation. Aim of the study was the reversal of hyperinflammation to improve pulmonary function, thus reducing ventilatory dependency and mortality. Patients older than 18 years hospitalized with COVID-19 pneumonia (demonstrated by chest X-ray or chest CT), a body temperature >37.3 °C, and either respiratory symptoms or hypoxia SpO_2_ < 93%, as well as a study specific CIS ≥ 10 were eligible. Patients with active tuberculosis, sepsis, uncontrolled bacterial, fungal, viral, or other infection (besides SARS-CoV-2 virus), or long-term use of oral anti-rejection or immunomodulatory drugs including cytokine-directed agents such as anti-IL6 or anti-IL1R directed antibodies (i.e., tocilizumab, anakinra), and pregnant or breastfeeding women were excluded from study participation. Patients with severe liver impairment, neutrophils <500/µL, platelets <50,000/µL), or hemoglobin <6 g/dl at screening were not eligible as well as patients with end-stage malignancy or pre-existing organ failure or/and with survival probability <6 months.

CIS was defined as the sum of the following parameters: i) chest X-ray or chest computed tomography (CT) with hypersensitivity pneumonitis, 3 points; ii) C-reactive protein >20x upper limit of normal (ULN), 2 points; iii) ferritin >2x ULN, 2 points; iv) triglycerides >1.5x ULN, 1 point; v) IL-6 > 3x ULN, 1 point; vi) fibrinogen >ULN, 1 point; vii) total white blood cell count >ULN, 1 point; viii) lymphocytes <1.1/μl, 2 points; body temperature >38.5 °C, 2 points; ix) coagulation disorder: D-dimer >ULN and/or activated partial thromboplastin time (aPTT) >ULN, 1 point (adapted from ref. [26]).

**Fig. 1:**
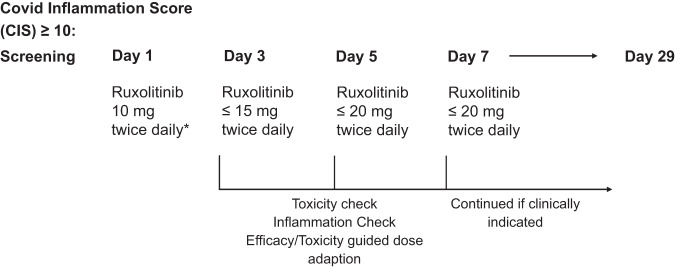
Study design. Patients with CIS (Covid Inflammation Score) ≥10 were eligible for treatment with ruxolitinib. Starting dosage was 10 mg twice daily (* dose adaptation to 5 mg twice daily permitted according to baseline organ function). If CIS was stable or rising on days 3 or 5 the dosage was escalated to 15 or 20 mg twice daily. In case of decreasing CIS (at least 25%), the dose level was maintained. Endpoint was CIS at day 7. Extension of study treatment up to day 29 was permitted weighing risks and benefit.

Patients were treated with ruxolitinib at a dose of 10 mg twice daily in addition to standard of care therapy for a duration of minimum 7 days with clinical and/or radiographic response assessment. Inflammation assessment was performed every other day (days 3, 5, 7) using the CIS. A response-guided predefined individual dose escalation scheme was implemented. In patients with unaffected (i.e., <25% change of CIS) or increasing CIS > 25% dose escalation by 5 mg twice daily steps was permitted at the investigator´s discretion up to a dose of 20 mg twice daily. Treatment could be extended up to 28 days if clinically indicated. Primary endpoint of the study was the overall response rate in reversal of hyperinflammation at day 7 compared to baseline. Secondary endpoints were duration of assisted oxygenation dependency (invasive/non-invasive ventilation or high-flow oxygen support), radiological response (reversal of pulmonary COVID-signs, lung X-ray/CT scan), day 15 clinical status, and day 15 and 28 mortality (Fig. 1).

The study was approved by the institutional review boards of participating institutions. Informed consent was obtained from patients or guardians.

### Study specific assessments

Clinical symptoms, radiological results, hematology, and routine clinical chemistry were assessed in all patients on-site in the participating institutions. Paired serum cytokine levels were measured in a subgroup of patients on days 0 and 7 after start of ruxolitinib. These analyses were carried out centrally using particle immunoassay (MILLIPLEX® Immunology Multiplex Assay, Merck Millipore, Burlington, MA, USA) and the Luminex multiparameter technology for quantification (LUMINEX® 200^TM^, Luminex, Austin, TX, USA) according to the manufacturers’ instructions by IMD, Institut für Medizinische Diagnostik GbR, IMD, Berlin-Potsdam, Germany.

For the assessment of adverse events all patients who received at least one dose of the investigational drug were considered. Adverse events were recorded using the Common Terminology Criteria for Adverse Events version 5 (CTCAE) during the ongoing study treatment and on days 15 and 29.

### Statistics

Primary endpoint of the study was the change in the CIS value from baseline (day 0) on days 3, 5, and 7. Individual score reduction of at least 25% was expected in more than 20% of the patients. Therefore, the null hypothesis (H0) was predefined as “≤20% of patients experience a 25% score reduction within 7 days” and was tested against the alternative hypothesis (H1) “>20% of patients experience a 25% score reduction within 7 days”. For the sample size estimation, the two-stage design of O’Brien-Fleming, allowing one interim analysis, was considered. The overall alpha level (one sided) was 0.025. An 80% power to reject H0 if the actual success rate was 30% was chosen. The rate of successes was estimated by the number of successes in the patient sample divided by its total sample size *n*.

Dynamics of cytokine and CIS levels were assessed by the paired Wilcoxon test. For the comparison of frequencies of cytokine elevation at baseline and day 7, the McNemar test was used. Apart from the primary endpoint, *p*-values below 0.05 were considered statistically significant.

## Results

**Fig. 2:**
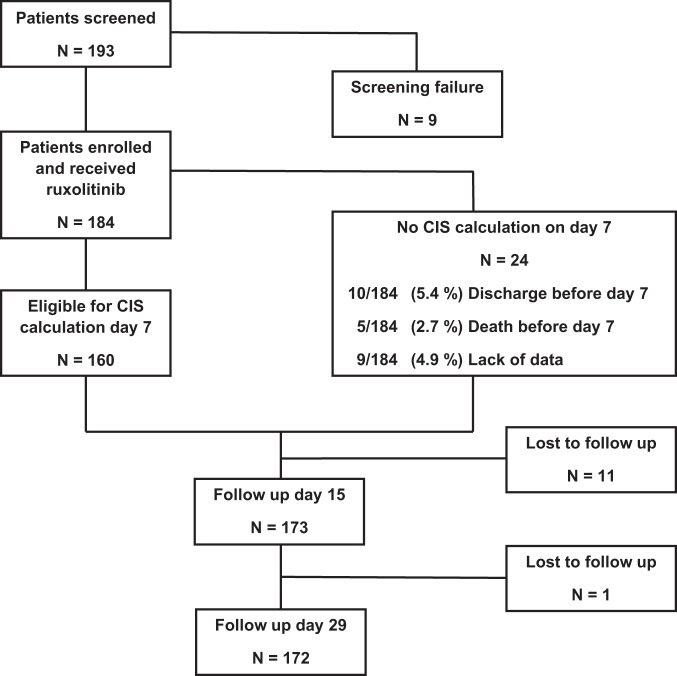
Trial profile. 193 patients were screened, of whom 184 patients were enrolled and subsequently treated with ruxolitinib in addition to standard of care. In 24 patients, CIS on day 7 was not available due to early discharge (5.4%), death before day 7 (2.7%), or lack of data for exact CIS calculation (4.9%). In 160 patients CIS was available on day 7, follow up on days 15 and 29 was recorded for 173 and 172 patients, respectively; while 12 patients were lost to follow up until day 29.

In seven institutions, 193 patients were screened for participation, of whom 184 (72.8% male; median age 62 years, range 25–90) were recruited and treated (Fig. 2) from April 2020 to June 2021. Median CIS at baseline was 12 points (range, 10–16). In two patients, exact CIS could not be calculated due to missing data, but CIS levels were ≥10 based on the parameters available. At inclusion, all patients showed bi-pulmonary pneumonitis, 35.3% of patients required insufflation of oxygen, 30.4% non-invasive and 28.3% invasive ventilation. Obesity (Body mass index >30 kg/m^2^) was present in 35.3% of the patients and 21.7% had a history of smoking. Charlson Comorbidity index (CCI) was median 2, 10.9% of patients had no comorbidity, 29.3% showed >4 comorbidities. The most common comorbidities comprised arterial hypertension, diabetes mellitus, and chronic lung disease. Median WHO 7 point scale, adapted from WHO recommendation for clinical trials [27], was 5 (range, 3–6), median Richmond Agitation Sedation Scale (RASS) [28] was −4 (range, −5–−2), median National Early Warning Score (NEWS2) [29] was 8 (range, 1–19). Median oxygen saturation was 93% (range, 72–100), 171 patients (92.9%) required oxygen support, 56 (30.4%) invasive ventilation (Table 1).

**Table 1:** Patients’ baseline characteristics and risk factors.

Baseline parameter	Median (range) or *n*/*N* (%)
Age, years	62 (25–90)
Age >80 years	21/184 (11.4)
Sex, male	134/184 (72.8)
Smoking history	40/184 (21.7)
Body mass index, kg/m^2^	28.2 (18.5–51.6)
Charlson Comorbidity Index	2 (0–12)
Most common comorbidities	
Arterial hypertension	95/184 (51.6)
Diabetes mellitus	38/184 (20.7)
Chronic lung disease	32/184 (17.4)
Bi-pulmonary pneumonitis	184/184 (100)
Respiratory rate	24/min (range 10–55)
Oxygen saturation, %	93 (72–100)
Heart rate ≥ 100/min	30/184 (16.3)
WHO 7 point scale [27]	5 (3–6)
3 points	11/182 (6.0)
4 or 5 points	119/182 (65.4)
6 points	52/182 (28.6)
RASS [28]	−4 (−5–−2)
NEWS2 [29]	8 (1–19)

*RASS* Richmond Agitation-Sedation Scale, *NEWS2* National Early Warning Score 2.

### Treatment and clinical efficacy

Ruxolitinib was applied over a median of 14 days (range, 2–31), median average daily dose was 22.6 mg. Median cumulative dose was 272.5 mg. Response guided dose escalation (in case of inadequate CIS reduction compared to baseline) on days 3 and 5 was conducted in 24.5 and 16.8% of the patients, respectively. Standard of care treatment comprised corticosteroids in 94.6%, remdesivir in 40.2%, and combination of corticosteroids and remdesivir in 38.6% of patients.

**Fig. 3:**
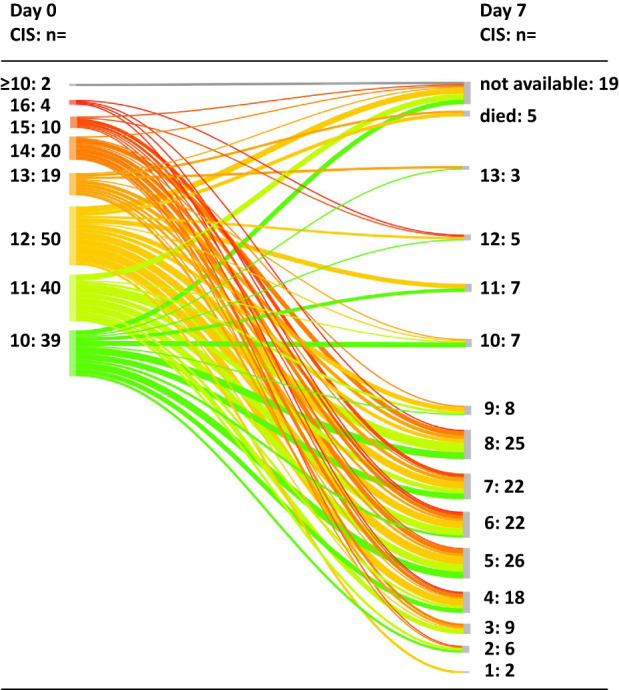
Initial dynamics of the Covid Inflammation Score (CIS) between days 0 and 7. At baseline, all patients showed CIS levels ≥10.

Median duration of the hospital stay was 18 days (range, 2–101). 75.7% of patients required intermediate or intensive care management for a median of 17.5 days. In 176 patients (97.2%), supplemental oxygen was required for a median of 8 days. Non-invasive and invasive ventilation was needed in 59.8% and 41.8 of patients for a median of 5 and 17 days, respectively. (Table 2) 130/184 (70.7%) of patients (95% confidence interval 64–77%) achieved an at least 25% reduction of the CIS score at day 7. Therefore, the null hypothesis was rejected, the primary endpoint of the study was met with ≥20% of patients achieving this result (α = 0.02498, *p* < 0.0001) at the final analysis. 43.4% showed a reduction of at least 50% (Table 3, Fig. 3). Six patients died from COVID-19 associated multiorgan failure within the first 7 days after initiation of ruxolitinib treatment, of whom five had baseline CIS ≥12 points.

**Table 2:** Individual course of treatment.

Parameters during the course of therapy	Median (range) or *n*/*N* (%)
Interval admission to first dose ruxolitinib, days	1 (0–32)
Duration ruxolitinib treatment, days	14 (2–31)
Cumulative dose, mg	272.5 (20–1085)
Mean dose per treatment day, mg	19.3 (9.5–36.8)
Dose adjustment on day 3	45/184 (24.5)
Dose adjustment on day 5	31/184 (16.8)
Dose adjustment at other times	42/184 (22.8)
Other treatments	
Corticosteroids	175/184 (95.1)
Remdesivir	74/184 (40.2)
Corticosteroids + remdesivir	71/184 (38.6)
Duration of hospitalization, days	18 (2–101)
ICU/IMC management	137/181 (75.7)
Duration, days	17.5 (2–75)
Supplemental oxygen	176/181 (97.2)
Duration, days	8.0 (1–44)
Non-invasive ventilation (NIV) n/N (%)	110/184 (59.8)
Duration, days	5 (1–26)
Invasive ventilation	77/184 (41.8)
Duration, days	17 (1–61)

*ICU* Intensive care unit, *IMC* Intermediate care unit.

**Table 3:** Dynamics of the COVID-19 inflammation score.

COVID Inflammation Score (CIS)	
	**Median (range);** ***n*/*N*** **(%)**
Baseline	12 (10–16); 182/184 (98.9)
Day 3	8 (2–14); 179/184 (97.3)
Day 5	7 (2–15); 173/184 (94.0)
Day 7	6 (1–13); 160/184 (87.0)
**CIS reduction between days 0 and 7**	***n*/*N*** **(%)**
≥25% reduction of CIS	130/184 (70.7)
≥50%	80/184 (43.4)
<25%	30/184(16.3)
No change or increase of CIS	13/184 (7.1)

Total white blood cells and lymphocyte counts, as well as triglyceride levels increased, ferritin, C-reactive protein and fibrinogen levels decreased significantly between days 0 and 7 (Supplementary Table S1).

Follow up data on days 15 and 29 are available in 173 and 172 patients, respectively. Twelve patients were lost to follow up or withdrew consent after hospital discharge. Mortality rate was 11.0% (19/173 patients) and 19.2% (33/172 patients) on days 15 and 29 after initiation of study treatment, respectively.

### Cytokine levels

**Fig. 4:**
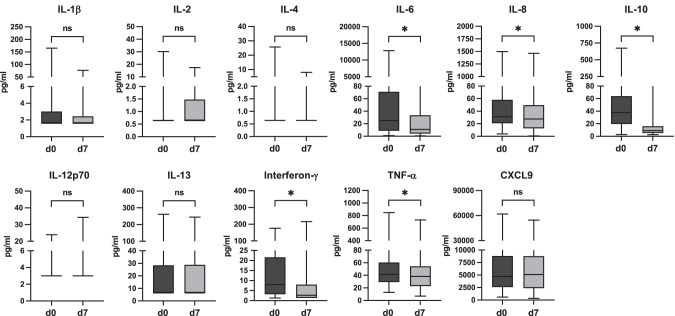
Dynamics of cytokine levels between baseline (day 0) and day 7 (*n* = 91). Significant changes are indicated by an asterisk (*). Abbreviations: IL Interleukin, TNF-α tumor necrosis factor α, CXCL9 CXC-Ligand 9, ns not significant. Reference ranges: IL-12 p70, IFN-γ, IL-10, IL-1β, IL-2, IL-4, IL-6: below 3.2 pg/ml each; IL-13, TNF-α: below 6.4 pg/ml each; IL-8: below 33.3 pg/ml; CXCL9: 265–2183 pg/ml.

Paired cytokine levels at baseline and on day 7 were assessed in 91 patients with comparable distribution of baseline characteristics and CIS as the study cohort (Supplementary Table S2). Elevation above upper limit of normal was observed for IL-10, IL-6, and TNF-α in nearby all patients (95.6%, 92.3%, and 98.9%, respectively), less often for Interferon-γ and CXCL9 (74.7% and 78.0%, respectively) (Supplementary Table S3). At baseline, absolute levels were scattered over a wide range. Median levels above the upper limit of normal were detected for Interferon-γ, IL-10, IL-6, CXCL9, and TNF-α. Between baseline and day 7, a significant decrease of absolute cytokine levels was revealed for Interferon-γ, IL-6, 8, and 10, as well as TNF-α (Table 4, Fig. 4). Comparing relative changes of elevation above different degrees of upper limit of normal, a significant decrease between baseline and day 7 was demonstrated for Interferon-γ, IL-6, and IL-10 at all degrees. For IL-8 and TNF-α, relative changes were not significant in contrast to changes in absolute levels.

**Table 4:** Cytokine levels in paired samples at baseline and day 7 after start of ruxolitinib therapy (*n* = 91).

	IL-12 p70		IFN-γ		IL-10		IL-13		IL-1β	
Reference range	<3.2 pg/ml		<3.2 pg/ml		<3.2 pg/ml		<6.4 pg/ml		<3.2 pg/ml	
	d0	d7	d0	d7	d0	d7	d0	d7	d0	d7
Median	3	3	8.0	2.67	37.8	9.19	6.4	6.4	1.6	1.6
*Mean*	*3.9*	*3.9*	*16.0*	*9.3*	*57.1*	*15.2*	*26.1*	*23.9*	*5.8*	*4.6*
Min.	3	3	1.3	1.3	2.6	2.6	6.4	6.4	1.6	1.6
Max.	23.9	34.3	176	216	672	147	262	245	165	76.5
*P*	0.391		**<0.001**		**<0.001**		0.188		0.264	

	IL-2		IL-4		IL-6		IL-8		CXCL9		TNF-α	
Reference range	<3.2 pg/ml		<3.2 pg/ml		<3.2 pg/ml		<33.3 pg/ml		265–2183 pg/ml		<6.4 pg/ml	
	d0	d7	d0	d7	d0	d7	d0	d7	d0	d7	d0	d7
Median	0.64	0.64	0.64	0.64	25.1	10.9	31.3	27.5	4723	5093	40.2	38.3
*Mean*	*1.83*	*1.58*	*1.55*	*0.95*	*251.2*	*44.4*	*93.5*	*59.7*	*6700*	*6691*	*61.3*	*49.7*
Min.	0.64	0.64	0.64	0.64	1.01	0.64	3.72	0.64	593	333	6.4	7
Max.	30.2	17.4	25.7	8.01	12806	706	1498	1463	61779	54630	847	731
*p*	0.591		0.119		**<0.001**		**0.032**		0.672		**0.015**	

*IFN* interferon, *IL* interleukin, *TNF-α* tumor necrosis factor alpha. *p*-values indication significant differences in bold.

### Adverse events

Two-hundred and twelve grade 1–5 adverse events (AEs) were reported, 54.3% of patients were affected. Most common AEs of all grades were sleeping problems (18.5%), weight loss (15.2%), elevated liver enzymes (8.7%), superinfections to COVID-19 associated pneumonitis (7.1%), bleeding complications (3.3%), and electrolyte imbalance (3.3%). These AEs were mostly graded 1 or 2 and reversible. Grade 3 or 4 adverse AEs of special interest were elevated liver enzymes (0.5%), infections (3.8%), and bleeding complications (1.1%).

Apart from elevation of liver enzymes grade 1 or 2 the relationship was recorded as unlikely or not related. Six bleeding complications were recorded, of which two were assessed grade 3 or 4. Adverse events of higher grades were documented in the severely ill study population, they comprised electrolyte imbalance, bleeding complications, (super-)infections and elevated liver enzymes. No grade 3–5 adverse event was classified as related to ruxolitinib. In total, 44 patients (23.9%) died, all without reported association to study drug.

## Discussion

The RuxCoFlam trial demonstrated a high efficacy of ruxolitinib in addition to standard of care in resolving hyperinflammation in a large cohort of COVID-19 patients with severe infection, defined by a CIS ≥ 10. The primary endpoint of the study represented the expectations at the time of the study design and was defined as individual score reduction of at least 25% in more than 20% of the patients within 7 days of therapy. The actual data exceeded the expectations significantly. Ruxolitinib was well tolerated, severe drug related side effects were not observed.

The need for the RuxCoFlam trial emerged during the early stage of the pandemic, when the principle of anti-inflammatory treatment in COVID-19 gained evidence. Fundamentally, the Randomized evaluation of Covid-19 therapy (Recovery) trial (NCT04381936) [30] demonstrated a benefit of low dose glucocorticoids, which was suggested standard of care in 2020. A strategy for treatment of COVID-19 hyperinflammation beyond low dose glucocorticoids was required urgently and thus targeting proinflammatory signaling was investigated intensively [31]. Inhibition of specific cytokines with a key role in hyperinflammation was the approach chosen in several trials. There was a conclusive theoretical basis for targeting IL-6 by tocilizumab in COVID-19 with positive early case reports and small series [32–34]. Confirmation emerged from a sub-study of the Recovery trial. The usage of tocilizumab was stratified by severity of COVID-19; only those patients with signs of advanced systemic inflammation (hypoxia and C-reactive protein ≥75 mg/L) were randomized and treated with tocilizumab in addition to glucocorticoids. Tocilizumab led to improved survival and favorable clinical outcomes [35]. In contrast no benefit in intubation rate or death was shown in a large review on general use of tocilizumab in COVID-19 [36] emphasizing the need for preselection. Antagonism of IL-1α/β by anakinra in COVID-19 patients at risk of developing respiratory failure showed clinical and short-term survival benefit [7]. Beneficial effects of TNF blockade were reported in an early serial case report [37] and a controlled trial suggesting a stronger effect of namilumab than infliximab [38].

Upregulation of cytokines in hyperinflammation is complex, multifactorial and may therefore not be silenced on the level of suppression of single cytokines. Hence, by targeting the JAK/STAT pathway a common route and effector of multiple cytokines with central role in COVID-19 associated hyperinflammation has been suggested as a promising strategy [16, 39]. Several inhibitors were tested, and baricitinib was approved in 2021 for treatment of COVID-19 [17, 18]. Initial concerns on the use of baricitinib with regard to potential thromboembolic complications in the prothrombotic state of COVID-19 [40, 41] had not been confirmed. [17] On balance, effective inhibition of inflammation and short-term use did not result in an excess incidence of thromboembolic events. [40] However, the application was withdrawn by the manufacturer in 2022 [42].

The inhibitor of the Janus kinases 1 and 2 ruxolitinib is currently approved for primary and certain secondary forms of myelofibrosis, hydroxyurea resistant polycythemia vera, and for graft versus host disease (GvHD) in steroid refractory patients [43–45]. Its high effectiveness, also beyond the use of glucocorticoids was demonstrated in other conditions that go along with cytokine release such as hemophagocytic lymphohistiocytosis (HLH) [46, 47]. These properties could be transferred to COVID-19. In a study on COVID-19 pneumonia, ruxolitinib appeared to be safe in COVID-19 patients, with clinical benefits observed in terms of decrease in a predefined 8-point ordinal scale and pro-inflammatory state [25]. Moreover, in a compassionate use program, ruxolitinib rapidly reduced the systemic inflammation, which accompanied COVID-19, thereby improving respiratory function and reducing the need of oxygen support [24]. Kelmenson and Cron defined the question of optimal timing of cytokine inhibition within the course of COVID-19 as crucial [14]. Concerning ruxolitinib in COVID-19 the numerous, parallel introduced trials can be interpreted as three approaches to clarify this question. i) Early use of ruxolitinib in COVID-19 to maximize the number that benefits potentially. ii) Use of ruxolitinib in very advanced stage, and iii) an approach that considers the individual stage of hyperinflammation as trigger for ruxolitinib as introduced in the RuxCoFlam trial. The RUXCOVID trial (NCT04362137) was a multinational, randomized, double-blind, phase 3 study of ruxolitinib plus standard of care versus placebo plus standard of care in patients within early stage of COVID-19. Precondition for treatment was hospitalization, patients in advanced stages requiring intensive care or mechanical ventilation were excluded. In this cohort, treatment was randomly assigned (2:1) to oral ruxolitinib 5 mg twice daily or placebo for 14–28 days. The primary endpoint was a composite of death, respiratory failure, or intensive care requirement by day 29.  Ruxolitinib did not show any benefit in the overall study population. The impact of the low-dose therapy remains unclear [19]. From our perspective, the proposed CIS-based preselection of patients in need for enhanced cytokine-directed anti-inflammatory treatment fits well to a number of negative trials devoid of inflammation-based treatment decisions, and to the positive usage of tocilizumab in the Recovery trial [35].

In contrast, Neubauer et al. treated very advanced staged COVID-19 patients with acute respiratory distress syndrome with higher doses of ruxolitinib (10–15 mg twice daily) and concluded potential efficacy at this stage (NCT04359290) [20, 21]. This could not be reproduced in the RUXCOVID-DEVENT trial (NCT04377620) in which exclusively COVID-19 patients suffering from acute respiratory distress syndrome (ARDS) with requirement of mechanical ventilation received ruxolitinib [22]. In advanced stages of COVID-19 disease, despite positive effects on hyperinflammation, ruxolitinib might not be able to reverse manifest organ damage.

The RuxCoFlam trial focused on the condition of hyperinflammation as treatment selection criteria, which was defined and quantified with the recently introduced COVID-19 inflammation score (CIS) [26]. By CIS-based stratification, a rational preselection strategy was offered. Ruxolitinib was applied to a cohort of critical ill COVID-19 patients with defined and critical hyperinflammation, requiring intensive care support in the majority. Most patients were treated effectively with the starting dose of 10 mg twice daily ruxolitinib in addition to dexamethasone without need for dose escalation. This dose was chosen based on the experience in GvHD and HLH [45, 47, 48].

The cohort of patients treated in RuxCoFlam is comparable to the dexamethasone arm in the Recovery trial. Here, 2104 patients, 61% in need of oxygen and 15% with mechanical ventilation were treated. Despite higher rate of initial mechanical ventilation in the RuxCoFlam trial, 4-week-mortality was comparable with 19.1% vs 22.8% in RuxCoFlam vs Recovery, respectively [30]. However, limitations of cross-trial comparisons should be considered.

Within seven days of treatment laboratory parameters and clinical signs of hyperinflammation reversed significantly. Within the trial, cytokine levels were measured to obtain evidence on the effective inhibition of hyperinflammatory pathways with ruxolitinib in vivo. However, hyperinflammation was defined on clinical parameters; the analysis of the predictive role of individual cytokine levels was not the aim of this study. Levels of cytokines involved in COVID-19 hyperinflammation declined rapidly: Interferon γ is a type II interferon that triggers antiviral and adaptive immune responses [49, 50], and is upregulated in COVID-19 [51].

The primary role of TNF-α is in the regulation of immune cells. It is able to induce fever, apoptotic cell death, cachexia, and inflammation, and inhibits viral replication. Both Interferon-γ and TNF-α play a major role in HLH [52] and COVID-19 induced hyperinflammation suggesting the similarity of the pathogenesis of those diseases. Synergy of Interferon-γ and TNF-α has the capacity of inducing hyperinflammation by a positive feedback loop via hyperactivation of the JAK/STAT1 pathway demonstrated by Kandhaya-Pillai et al. in an in vitro model of endothelial cells in interaction with SARS-CoV-2 [53]. Using ruxolitinib, this interactive loop is disrupted and hyperinflammation terminated. The RuxCoFlam patients showed a significant downregulation of central cytokines IL-6 and IL-10 under treatment with ruxolitinib. IL-10 has been described as the ‘master regulator’ of immune responses through JAK/STAT [54]. In the presented approach influence of comedication with glucocorticoids and natural limitation of hyperinflammation cannot be excluded. However, the pattern of cytokine decline does reflect the major role of JAK/STAT inhibition.

Adverse events were frequently reported in the cohort of preselected severely ill patients. They were commonly attributed to the context of hyperinflammatory syndrome, consecutive organ failure and intensive supportive treatment. Typical side effects of ruxolitinib were in focus during the trial; however, severe cytopenias were not reported. Hence, myelosuppression may not represent a major problem in short-term application in patients with regular and not JAK-mutated hematopoiesis. Elevation of liver enzymes is a known side effect of ruxolitinib in long-term application. The reported low-grade events in the RuxCoFlam study were reversible in all patients. Withdrawal from study medication due to liver toxicity was not required in any patient. Bleeding complications were related to preexisting disease, comedication, and complications of the critical illness. Relationship to study medication was not observed.

In summary, hyperinflammation was resolved in most COVID-19 patients with ruxolitinib. The oral treatment was feasible in critically ill patients, application was safe without high grade or lasting adverse treatment associated reactions. Detecting the critical stage of hyperinflammation with COVID-19 inflammation score provides a tool for accurate preselection of COVID-19 patients and by that optimizes clinical benefit of ruxolitinib therapy.

## Supplementary information

Table S1

Table S2

Table S3

## Data Availability

Authors are committed to sharing access to patient-level data and supporting clinical documents with qualified external researchers upon request. These requests are reviewed and approved by an independent review panel based on scientific merit. All data provided are anonymized to respect the privacy of patients who have participated in the trial consistent with applicable laws and regulations.
