# Supplementary material for: The JAK1/2 inhibitor ruxolitinib in patients with COVID-19 triggered hyperinflammation: the RuxCoFlam trial
Source: Leukemia. 2023 Jul 28;37(9):1879–86. doi: 10.1038/s41375-023-01979-w (PMC10457200; doi:10.1038/s41375-023-01979-w)
Supplement: Supplementary file 1 — Table S1 [file 41375_2023_1979_MOESM1_ESM.docx]

**Supplemental Table S1**. Hematology and clinical chemistry.

| **Parameter** | **Reference range** | **Baseline Median (range)** | **Day 7 Median (range)** | **Median of change in %** | **p** |
| --- | --- | --- | --- | --- | --- |
| Leukocyte count, /nL | 4.4- 11.3 | 7.9 (1.6 – 38.7) | 10.0 (2.1 – 72.0) | + 26.5 | <0.001 |
| Lymphocyte count, /nL | 1.0-4.8 | 0.68 (0.05 – 32.6) | 1.24 (0.26 – 56.9) | + 89.2 | <0.001 |
| Ferritin, µg/L | 30-400 | 1737 (200 – 9500) | 1515 (110 – 5326) | - 20.4 | <0.001 |
| C-reactive protein, mg/L | < 5 | 151 (20 – 480) | 31 (1 – 267) | - 81.2 | <0.001 |
| Triglycerides, mmol/L | < 1.7 | 1.55 (0.58 – 6.0) | 2.1 (0.5 – 6.3) | + 22.2 | <0.001 |
| Fibrinogen, g/L | 2.4-4.9 | 5.9 (3.2 – 10.0) | 4.1 (0.67 – 8.0) | - 28.9 | <0.001 |
