# Supplementary material for: The JAK1/2 inhibitor ruxolitinib in patients with COVID-19 triggered hyperinflammation: the RuxCoFlam trial
Source: Leukemia. 2023 Jul 28;37(9):1879–86. doi: 10.1038/s41375-023-01979-w (PMC10457200; doi:10.1038/s41375-023-01979-w)
Supplement: Supplementary file 2 — Table S2 [file 41375_2023_1979_MOESM2_ESM.docx]

**Supplemental Table S2**.

Characteristics of 91 patients for whom cytokine dynamics between baseline and day 7 were assessed.

| Age, years | median (range) | 59 (29 – 88) |
| --- | --- | --- |
| CIS baseline  CIS 10 – 11 points  CIS 12 – 13 points  CIS 14 – 16 points | median (range)  n/N (%)  n/N (%)  n/N (%) | 12 (10 – 16)  39/89 (43.8 %)  30/89 (33.7 %)  20/89 (22.5 %) |
| WHO 7-point scale baseline, points | median (range) | 5 (range 3 – 6) |
| CIS Day 7  Reduction of at least 25 %  Reduction of at least 50 %  Reduction less than 25 % | median (range)  n/N (%)  n/N (%)  n/N (%) | 6 (1 – 13)  74/89 (83.1)  45/89 (50.6)  15/89 (16.9) |
| WHO 7-point scale baseline, points | median (range) | 5 (2 – 6) |
