# Supplementary material for: The JAK1/2 inhibitor ruxolitinib in patients with COVID-19 triggered hyperinflammation: the RuxCoFlam trial
Source: Leukemia. 2023 Jul 28;37(9):1879–86. doi: 10.1038/s41375-023-01979-w (PMC10457200; doi:10.1038/s41375-023-01979-w)
Supplement: Supplementary file 3 — Table S3 [file 41375_2023_1979_MOESM3_ESM.docx]

**Supplemental Table S3.** Comparison of frequencies of cytokine elevation at baseline and day 7.

| **IL-12 p70** | **> ULN** | **> 2x ULN** | **> 5x ULN** | **> 10x ULN** |
| --- | --- | --- | --- | --- |
| Baseline, n/N (%) | 15/91 (16.5) | 8/91 (8.8) | 3/91 (3.3) | 0/91 (0) |
| Day 7, n/N (%) | 14/91 (15.4) | 5/91 (5.5) | 2/91 (2.2) | 1/91 (1.1) |
| P | 1.000 | 0.453 | 1.000 | 1.000 |
|  | | | | |
| **IFN-γ** | **> ULN** | **> 2x ULN** | **> 5x ULN** | **> 10x ULN** |
| Baseline, n/N (%) | 68/91 (74.7) | 50/91 (54.9) | 26/91 (28.6) | 13/91 (14.3) |
| Day 7, n/N (%) | 42/91 (46.2) | 28/91 (30.8) | 9/91 (9.9) | 4/91 (4.4) |
| P | **<0.001** | **0.002** | **<0.001** | **0.022** |
|  | | | | |
| **IL-10** | **> ULN** | **> 2x ULN** | **> 5x ULN** | **> 10x ULN** |
| Baseline, n/N (%) | 87/91 (95.6) | 86/91 (94.5) | 73/91 (80.2) | 52/91 (57.1) |
| Day 7, n/N (%) | 79/91 (86.8) | 60/91 (65.9) | 23/91 (25.3) | 11/91 (12.1) |
| P | **0.021** | **<0.001** | **<0.001** | **<0.001** |
|  | | | | |
| **IL-13** | **> ULN** | **> 2x ULN** | **> 5x ULN** | **> 10x ULN** |
| Baseline, n/N (%) | 40/91 (44.0) | 32/91 (35.2) | 22/91 (24.2) | 10/91 (11.0) |
| Day 7, n/N (%) | 41/91 (45.1) | 34/91 (37.4) | 20/91 (22.0) | 8/91 (8.8) |
| P | 1.000 | 0.815 | 0.791 | 0.727 |
|  | | | | |
| **IL-1β** | **> ULN** | **> 2x ULN** | **> 5x ULN** | **> 10x ULN** |
| Baseline, n/N (%) | 19/91 (20.9) | 12/91 (13.2) | 6/91 (6.6) | 2/91 (2.2) |
| Day 7, n/N (%) | 19/91 (20.9) | 10/91 (11.0) | 5/91 (5.5) | 3/91 (3.3) |
| P | 1.000 | 0.727 | 1.000 | 1.000 |
|  | | | | |
| **IL-2** | **> ULN** | **> 2x ULN** | **> 5x ULN** | **> 10x ULN** |
| Baseline, n/N (%) | 9/91 (9.9) | 4/91 (4.4) | 3/91 (3.3) | 0/91 (0) |
| Day 7, n/N (%) | 9/91 (9.9) | 4/91 (4.4) | 1/91 (1.1) | 0/91 (0) |
| P | 1.000 | 1.000 | 0.500 | Not applicable |
|  | | | | |

| **IL-4** | **> ULN** | **> 2x ULN** | **> 5x ULN** | **> 10x ULN** |
| --- | --- | --- | --- | --- |
| **Baseline, n/N (%)** | 6/91 (6.6) | 4/91 (4.4) | 2/91 (2.2) | 0/91 (0) |
| **Day 7, n/N (%)** | 5/91 (5.5) | 1/91 (1.1) | 0/91 (0) | 0/91 (0) |
| **P** | 1.000 | 0.250 | 0.500 | Not applicable |
|  | | | | |
| **IL-6** | **> ULN** | **> 2x ULN** | **> 5x ULN** | **> 10x ULN** |
| **Baseline, n/N (%)** | 84/91 (92.3) | 78/91 (85.7) | 58/91 (63.7) | 38/91 (41.8) |
| **Day 7, n/N (%)** | 73/91 (80.2) | 61/91 (67.0) | 38/91 (41.8) | 23/91 (25.3) |
| **P** | **0.003** | **<0.001** | **0.003** | **0.015** |
|  | | | | |
| **IL-8** | **> ULN** | **> 2x ULN** | **> 5x ULN** | **> 10x ULN** |
| **Baseline, n/N (%)** | 44/91 (48.4) | 19/91 (20.9) | 10/91 (11.0) | 8/91 8.8) |
| **Day 7, n/N (%)** | 35/91 (38.5) | 17/91 (18.7) | 4/91 (4.4) | 2/91 (2.2) |
| **P** | 0.137 | 0.839 | 0.180 | 0.109 |
|  | | | | |
| **CXCL9** | **> ULN** | **> 2x ULN** | **> 5x ULN** | **> 10x ULN** |
| **Baseline, n/N (%)** | 71/91 (78.0) | 51/91 (56.0) | 15/91 (16.5) | 1/91 (1.1) |
| **Day 7, n/N (%)** | 75/91 (82.4) | 51/91 (56.0) | 17/91 (18.7) | 2/91 (2.2) |
| **P** | 0.556 | 1.000 | 0.845 | 1.000 |
|  | | | | |
| **TNF-α** | **> ULN** | **> 2x ULN** | **> 5x ULN** | **> 10x ULN** |
| **Baseline, n/N (%)** | 90/91 (98.9) | 90/91 (98.9) | 65/91 (71.4) | 16/91 (17.6) |
| **Day 7, n/N (%)** | 91/91 (100.0) | 86/91 (94.5) | 58/91 (63.7) | 12/91 (13.2) |
| **p** | 1.000 | 0.125 | 0.248 | 0.481 |

*Abbreviations; IL, interleukin; ULN, upper limit of normal*
